# High EGFR copy number predicts benefits from tyrosine kinase inhibitor treatment for non-small cell lung cancer patients with wild-type EGFR

**DOI:** 10.1186/1479-5876-11-90

**Published:** 2013-04-04

**Authors:** Fang Wang, Sha Fu, Qiong Shao, Yan-Bin Zhou, Xiao Zhang, Xu Zhang, Cong Xue, Jian-Guang Lin, Li-Xia Huang, Li Zhang, Wei-Min Zhang, Jian-Yong Shao

**Affiliations:** 1 State Key Laboratory of Oncology in South China, Sun Yat-sen University Cancer Center, Guangzhou, China; 2 Department of Molecular Diagnostics, Sun Yat-sen University Cancer Center, 651 Dongfeng Rd East, Guangzhou 510060, China; 3 Department of Internal Medicine and Pulmonary Medicine, First Affiliated Hospital, Sun Yat-Sen University, Guangzhou, China; 4 Department of Medicine Oncology, Sun Yat-sen University Cancer Center, Guangzhou, China; 5 Department of Oncology, General Hospital of Guangzhou Military Command, Guangzhou, China; 6 Lung Cancer Institute, Sun Yat-sen University, Guangzhou, China

**Keywords:** EGFR, Mutation, Copy number, Lung cancer

## Abstract

**Background:**

This study was designed to determine whether advanced non-small-cell lung cancer (NSCLC) patients with high copy number of epidermal growth factor receptor (EGFR) can benefit from treatment with EGFR-tyrosine kinase inhibitors (TKIs).

**Methods:**

EGFR gene copy number was assessed by fluorescence in situ hybridization (FISH) and EGFR mutations was tested using Luminex xTAG technology in 502 TKI-treated NSCLC patients. The association between both biomarkers and clinical benefit from EGFR-TKI were analyzed.

**Results:**

EGFR FISH + and EGFR mutations were significantly associated with higher response rates (37.2% and 43.7%, respectively), superior progression-free survival (PFS) (FISH+, 11.2 months; hazard ratio [HR], 0.51; 95% CI, 0.42 to 0.62; p < 0.001; mutation+, 11.7 months; HR, 0.37; 95% CI, 0.31 to 0.45; p < 0.001) and overall survival (OS) (FISH+, 30.2 months; HR, 0.51; 95% CI, 0.40 to 0.65; p < 0.001; mutation+, 30.2 months; HR, 0.45; 95% CI, 0.36 to 0.58; p < 0.001). In patients with wild-type EGFR, EGFR FISH + correlated with longer PFS than EGFR FISH- status (4.4 months *vs*. 2.0 months; HR, 0.56; 95% CI, 0.41 to 0.75; p < 0.001), so did amplification (5.0 months *vs*. 2.0 months; HR, 0.43; 95% CI, 0.24 to 0.76; p = 0.003). However, FISH + had no association with improved PFS in EGFR-mutated patients (HR, 0.77; 95% CI, 0.57 to 1.03; p = 0.076).

**Conclusions:**

**A** combined analysis of EGFR FISH and mutation is an effective predictor of EGFR-TKI therapy. Specifically, a high EGFR copy number may predict benefit from TKIs treatment for NSCLC patients with wild-type EGFR.

## Introduction

Increasing evidence indicates that activation of somatic mutations in the EGFR kinase domain (exons 18–21) [1,2] confers sensitivity to the EGFR TKIs, such as gefitinib and erlotinib for patients with advanced NSCLC. Several phase 3 randomized trials have shown that EGFR-TKIs offered significant benefits over standard chemotherapy in patients with EGFR mutation-positive tumors [3-7]. As an independent molecular subtype, the detection of EGFR mutations have been recommended in the National Comprehensive Cancer Network (NCCN) clinical practice guidelines in oncology (version 3.2011) to predict TKI sensitivity in clinical practice.

An increase in EGFR copy number may serve as a contributory mechanism for the activation of EGFR tyrosine kinase, and may trigger downstream oncogenic pathways [8,9]. A high EGFR copy number showed a trend toward poor prognosis in the absence of EGFR-TKI treatment [10,11]. Recent studies have shown that high EGFR gene copy number is associated with increased response rates to TKI therapy, as well as improved PFS [12,13] and OS[14-16]. Several studies have demonstrated that increased EGFR gene copy number and mutations display a high degree of overlap and the fluorescence in-situ hybridization-positive (FISH+) rate in patients with EGFR mutations was approximately 62.5% to 77.6% [3,17-20].

Although EGFR mutations can account for most of the objective responses to EGFR-TKIs therapy, the clinical benefits cannot only be explained by these mutations. Considering with mutant allele specific imbalance of oncogenes in tumor cells harboring gene mutation, copy number gain of EGFR usually occurred in the cells with an EGFR mutation [21]. It appears that the association between EGFR FISH + tumors and TKI sensitivity is due to coexisting EGFR mutations. In contrast to consistent reports of EGFR mutations correlating with improved response rates, reports regarding the predictive value of EGFR gene copy number have been inconsistent. Therefore, we sought to determine whether high EGFR copy number could be an alternative predictor for the efficacy of EGFR-TKIs in EGFR wild-type tumors.

In this study, we retrospectively detected EGFR mutations and gene copy number in order to evaluate the predictive value, alone or combined, for TKI efficacy and survival in TKI-treated patients.

## Methods

### Patient selection

This retrospective study included patients with histologically confirmed stage IIIb, stage IV, or recurrent NSCLC who received gefitinib or erlotinib treatment at any time during the course of their disease, between April 2004 and March 2011 at three Chinese institutions: Sun Yat-sen University Cancer Center, the First Affiliated Hospital of Sun Yat-sen University, and the Military General Hospital of Guangzhou. Patients were selected based on the following criteria: sufficient tumor tissue from primary or metastatic tumors obtained at the time of initial diagnosis for detection of EGFR mutations and FISH status, the presence of at least one measurable lesion according to the Response Evaluation Criteria In Solid Tumors (RECIST version 1.0) [22], and complete follow-up information (at least one evaluation before disease progression, more than three months after follow-up, or upon death). Patients were excluded if they had uncontrolled brain metastases or other primary cancers that were diagnosed either before or after NSCLC. Clinical follow-up information was obtained from the medical records of in-patients or out-patients, as well as telephone interviews. The study was approved by the Research Ethics Committee of the Sun Yat-sen University Cancer Center.

The medical history of each patient was documented by a retrospective chart review, which included age at diagnosis, gender, dates of diagnosis and death, postoperative disease recurrence, Eastern Cooperative Oncology Group (ECOG) performance status at the start of treatment with an EGFR-TKI, the number of previous chemotherapy regimens received, prior administration of a platinum-based drug, the EGFR-TKI administered (gefitinib or erlotinib), and subsequent treatment after progression. Tumor histology was classified according to the World Health Organization (WHO) criteria [23]. Clinical stage was based on the revised international staging system for lung cancer by the Union for International Cancer Control (UICC) [24] in 2009. Smoking status was categorized as ever or never (<100 lifetime cigarettes).

Gefitinib or erlotinib were administered orally (250 mg or 150 mg, respectively) once daily until disease progression, intolerable toxicity, or patient refusal. Clinical response was assessed every 3–10 weeks by radiologic examination (computed tomography or magnetic resonance imaging). Brain magnetic resonance imaging or radionuclide bone scans were added when brain or bone metastasis was suspected. The response was evaluated according to the RECIST criteria.

### DNA extraction and EGFR mutation detection

The QIAamp DNA FFPE Tissue Kit (Qiagen, Hilden, Germany) was used to extract DNA from paraffin-embedded tissues, and the operational tumor samples with histological control for the presence of tumor cells (> 70%) that was obtained by trimming the normal tissue and necrotic tissue.

EGFR mutations were analyzed by using The Surplex® EGFR Mutation Kit (Surexam Bio-Tech, Guangzhou, China) to screen for 22 mutations (Additional file 1: Table S1) of EGFR exons18–21 in an x-TAG liquidchip assay. The main procedures are listed as follows [25]: EGFR gene fragments were obtained by PCR containing 22 mutation sites; and the excess primers and dNTPs were removed by exonuclease I and alkaline phosphatase (EXO-SAP). The EXO-SAP-cleaned PCR product was subjected to an allele specific primer extension (ASPE) step where a universal tag was linked to a specific primer sequence complementary to EGFR. The ASPE products were hybridized to specific anti-tag probes that were pre-coated on the magnetic microspheres. The magnetic microspheres were then applied to the Luminex 200 (Luminex Corp., Austin, TX) and median fluorescence intensity was read.

### EGFR FISH assay

Gene copy number per cell was investigated by FISH using the LSI EGFR Spectrum Orange/CEP7 Spectrum Green probe (Vysis, Abbott Laboratories, Illinois, USA) according to a published protocol with minor modifications. Detailed FISH staining procedures are described in our previously published articles [26]. FISH signals for each locus-specific FISH probe were assessed under an Olympus BX51 TRF microscope (Olympus, Japan) equipped with a triple-pass filter (DAPI/Green/Orange, Vysis). FISH analysis was independently performed by pathologists who were blinded to the clinical characteristics and molecular variables of the patients. A scheme for classifying NSCLC tumors as EGFR FISH + and EGFR FISH- was developed at the University of Colorado, and has been used in multiple clinical studies. FISH results for NSCLC were determined according to a previous description [14,27,28]. Patients were classified into six FISH strata with an increasing number of EGFR gene copies per cell according to the frequency of tumor cells with a specific number of EGFR gene copies and chromosome 7 centromere: disomy (≤ 2 copies in > 90% of cells); low trisomy (≤ 2 copies in ≥ 40% of cells, 3 copies in 10%–40% of the cells, ≥ 4 copies in <10% of cells); high trisomy (≤ 2 copies in ≥ 40% of cells, 3 copies in ≥ 40% of cells, ≥ 4 copies in < 10% of cells); low polysomy (≥ 4 copies in 10% – 40% of cells); high polysomy (≥ 4 copies in ≥ 40% of cells); and gene amplification (defined by presence of tight EGFR gene clusters and a ratio of EGFR gene to chromosome of ≥ 2 or ≥ 15 copies of EGFR per cell in ≥ 10% of analyzed cells).

### Statistical analysis

PFS as a primary endpoint was calculated from the time of the first TKI treatment to the time of disease progression according to RECIST criteria [22], or unacceptable toxic effects. Secondary endpoints included the objective response rate (ORR), disease control rate (DCR) and OS. OS was calculated from the time of first TKI treatment to patient death from any cause or last contact. Differences in distribution of baseline characteristics between groups, ORR, and DCR, were evaluated by χ2 test. PFS, OS, and 95% confidence intervals (CIs) were calculated by Kaplan–Meier survival analysis. PFS and OS were compared between groups using the log-rank test. Cox proportional hazards models were used to evaluate independent predictive factors of each biological and clinical feature associated with survival. All statistical analyses were performed using SPSS 16.0 for Windows (SPSS Inc., Chicago, Illinois), and p < 0.05 was considered statistically significant.

## Results

### Patient characteristics

NSCLC tumors from 502 patients were detected for EGFR FISH and EGFR mutation status among the 889 patients who were treated with EGFR-TKI. Baseline and treatment characteristics are summarized in Additional file 2: Table S2. Among the patients, 139 (27.7%) achieved an objective tumor response, 199 (39.6%) had stable disease, and 164 (32.7%) had progressive disease. Sixty-three patients (12.5%) continued receiving TKIs (median duration, 4.75 months; range, 0.7 to 34.5 months) after assessed with disease progression, and 42 patients (8.3%) received the other TKI as subsequent treatment. The last follow-up date was April 26, 2012 and median follow-up was 14.9 months (range, 1 to 81.3 months). At the time of analysis, 73 patients (14.5%) were still receiving TKIs. In total, 280 (55.8%) deaths occurred.

### EGFR FISH and TKI efficacy

**Figure 1:**
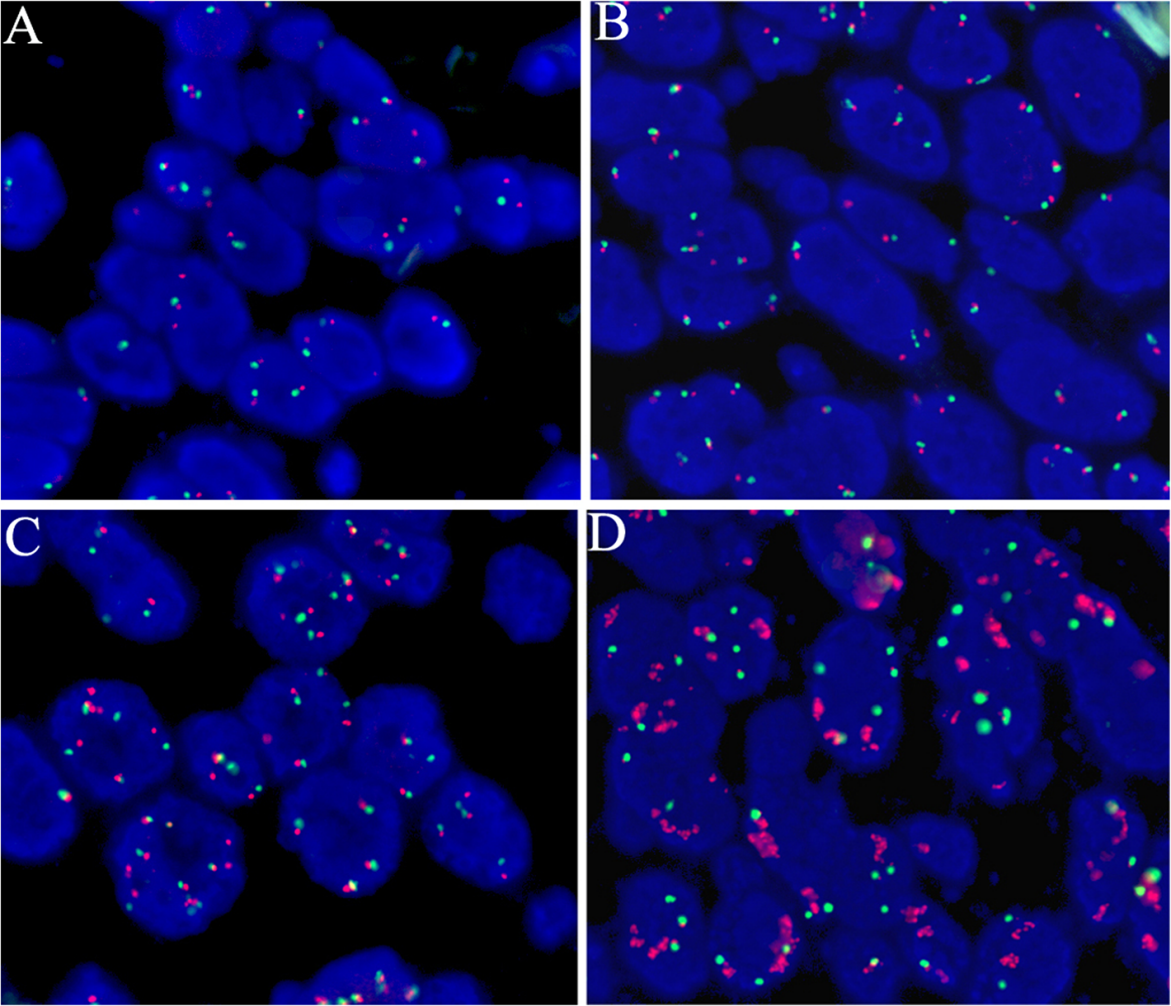
**Fluorescent in situ hybridization for epidermal growth factor receptor (EGFR) (orange signal) and centromere 7 (green signal) showing low (disomy = A; high triomy = B) copy number per cell (EGFR-FISH negative), high (high polysomy = C; gene amplification = D) copy number per cell (EGFR-FISH positive) (A-D, 1,000×)**.

The distribution of EGFR-FISH categories was as follows: disomy was present in 166 patients (33.1%), low trisomy in 29 (5.8%), high trisomy in 9 (1.8%), low polysomy in 72 (14.3%), high polysomy in 135 (27.5%), and gene amplification in 91 (18.1%) (Figure 1A-D). Two hundred and twenty-six patients (45.0%) were categorized as EGFR FISH + (high EGFR copy number), and 276 patients (55.0%) were characterized as EGFR FISH- (low EGFR copy number). FISH + patients were more likely to be female (p = 0.007) and non-smokers (p = 0.030) (Additional file 3: Table S3). There were significant differences in ORR (p < 0.001), DCR (p < 0.001), PFS (11.2 moths *vs*. 3.0 months; HR, 0.51; 95% CI, 0.42 to 0.62; p < 0.001), and OS (30.2 moths *vs*.17.2 months; HR, 0.51; 95% CI, 0.40 to 0.65; p < 0.001) between EGFR FISH + and EGFR FISH- patients (Tables 1, 2).

**Table 1:** Survival analysis and clinical response to EGFR-TKI treatment in relation to biomarkers of EGFR FISH and mutation in all patients

**EGFR Status**	**PFS, months**		**p**^#^	**OS, months**		**p**^**#**^	**Tumor Response**					p^*^
	**median**	**95%CI**		**median**	**95%CI**		**ORR (%)**	**Non-ORR (%)**	p^*^	**DCR (%)**	**Non-DCR (%)**	
FISH + (*n* = 226)	11.2	8.9-13.5	< 0.001	30.2	23.8-36.5	< 0.001	84 (37.2)	142 (62.8)	< 0.001	192 (85.0)	34 (15.0)	< 0.001
FISH- (*n* = 276)	3.0	2.1-3.8		17.2	14.6-19.7		55 (19.9)	221 (80.1)		146 (52.9)	130 (47.1)	
EGFR Mutation + (*n* = 252)	11.7	10.0-13.3	< 0.001	30.2	24.8-35.6	< 0.001	110 (43.7)	142 (56.3)	< 0.001	227 (90.1)	25 (9.9)	< 0.001
EGFR Mutation – (*n* = 247)	2.3	1.8-2.7		15.4	13.2-17.7		27 (10.9)	220 (89.1)		108 (43.7)	139 (56.3)	
Mutation+/FISH + (*n* = 163)	12.9	10.0-15.9	0.075	35.9	27.4-44.3	0.051	72 (44.2)	91 (55.8)	0.821	149 (91.4)	14 (9.6)	0.339
Mutation+/FISH- (*n* = 89)	9.3	6.1-12.6		27.9	23.4-32.3		38 (42.7)	51 (57.3)		78 (87.6)	11 (12.4)	
Mutation-/FISH + (*n* = 62)	4.4	2.3-6.4	< 0.001	25.0	14.1-35.9	0.009	11 (17.7)	51 (82.3)	0.047	42 (67.7)	20 (32.3)	< 0.001
Mutation-/FISH- (*n* = 185)	2.0	1.7-2.3		14.2	11.5-16.9		16 (8.6)	169 (91.4)		66 (35.7)	119 (64.3)	

Abbreviation: *FISH*, Fluorescent in situ hybridization; *ORR*, Objective response rate; *DCR*, Disease control rate; *PFS*, Progression-free survival; *OS*, Overall survival.

^#^p values (two-sided) calculated using the log-rank test.

^*^p values (two-sided) calculated using Pearson’s chi-square test.

**Table 2:** Cox proportional regression analysis for progression-free survival and overall survival

**Variable**	**PFS**				**OS**	
	**Univariate**		**Multivariate**		**Univariate**	
	**HR**	**p**	**HR**	**p**	**HR**	**p**
	**(95% CI)**		**(95% CI)**		**(95% CI)**	
Age	0.96	0.657			1.11	0.374
(<57 *vs*. γ 57)	(0.79 to 1.16)				(0.88 to 1.41)	
Gender	0.63	< 0.001	0.87	0.310	0.54	< 0.001
(female *vs.* male)	(0.52 to 0.77)		(0.66 to 1.14)		(0.42 to 0.69)	
Smoking status	1.57	< 0.001	1.25	0.102	1.67	< 0.001
(never *vs.* ever)	(1.29 to 1.92)		(0.96 to 1.64)		(1.31 to 2.12)	
Histology	1.37	0.009	0.94	0.673	1.25	0.133
(ADC *vs.* non-ADC)	(1.08 to 1.75)		(0.72 to 1.24)		(0.93 to 1.68)	
Stage	1.00	0.976			0.87	0.190
(IIIb *vs*. IV)	(0.83 to 1.20)				(0.70 to 1.07)	
ECOG	2.45	< 0.001	2.53	< 0.001	4.43	< 0.001
(0, 1 *vs.* 2, 3)	(1.95 to 3.08)		(1.97 to 3.24)		(3.33 to 5.89)	
LINE	1.32	0.006	1.06	0.618	1.40	0.006
(first-line *vs.*γ second-line)	(1.09 to 1.61)		(0.85 to 1.32)		(1.10 to 1.78)	
EGFR-TKI	1.14	0.201			1.14	0.312
(gefitinib *vs.* erlotinib)	(0.93 to 1.40)				(0.89 to 1.46)	
EGFR mutation	0.37	< 0.001	0.42	<0.001	0.45	< 0.001
(wild-type *vs*. mutation)	(0.31 to 0.45)		(0.34 to 0.53)		(0.36 to 0.58)	
EGFR FISH	0.51	< 0.001	0.61	<0.001	0.51	< 0.001
(FISH- *vs*. FISH+)	(0.42 to 0.62)		(0.49 to 0.76)		(0.40 to 0.65)	

Abbreviation: *ADC*, Adenocarcinoma; *ECOG*, Eastern cooperative oncology group; *TKI*, Tyrosine kinase inhibitors; *FISH*, Fluorescent in situ hybridization.

### EGFR mutation and TKI efficacy

Two hundred and fifty-seven mutations of EGFR gene were detected in 252 (50.5%) of the 499 analyzed patients. All the mutations detected in this study were shown in Additional file 1: Table S1. 140 patients had a deletion in exon 19,104 patients had an exon 21 missense mutation, three had an exon 18 missense mutation and five had combined mutations. Patients with EGFR mutations had higher ORRs (p < 0.001), DCRs (p < 0.001), and improved PFS (11.7 months; HR, 0.37; 95% CI, 0.31 to 0.45; p < 0.001) and OS (30.2 months; HR, 0.45; 95% CI, 0.36 to 0.58; p < 0.001) compared to patients with wild-type EGFR (Tables 1, 2).

In multivariate analysis, EGFR mutations (HR, 0.42; 95% CI, 0.34 to 0.53; p < 0.001) and a high EGFR copy number (HR, 0.61; 95% CI, 0.49 to 0.76; p < 0.001) were independent predictors of a longer PFS, in addition to an ECOG performance status of 2 and 3 (HR, 2.53; 95% CI, 1.97 to 3.24; p < 0.001) (Table 2).

### Efficacy of TKI in patients with EGFR FISH and EGFR mutations

**Figure 2:**
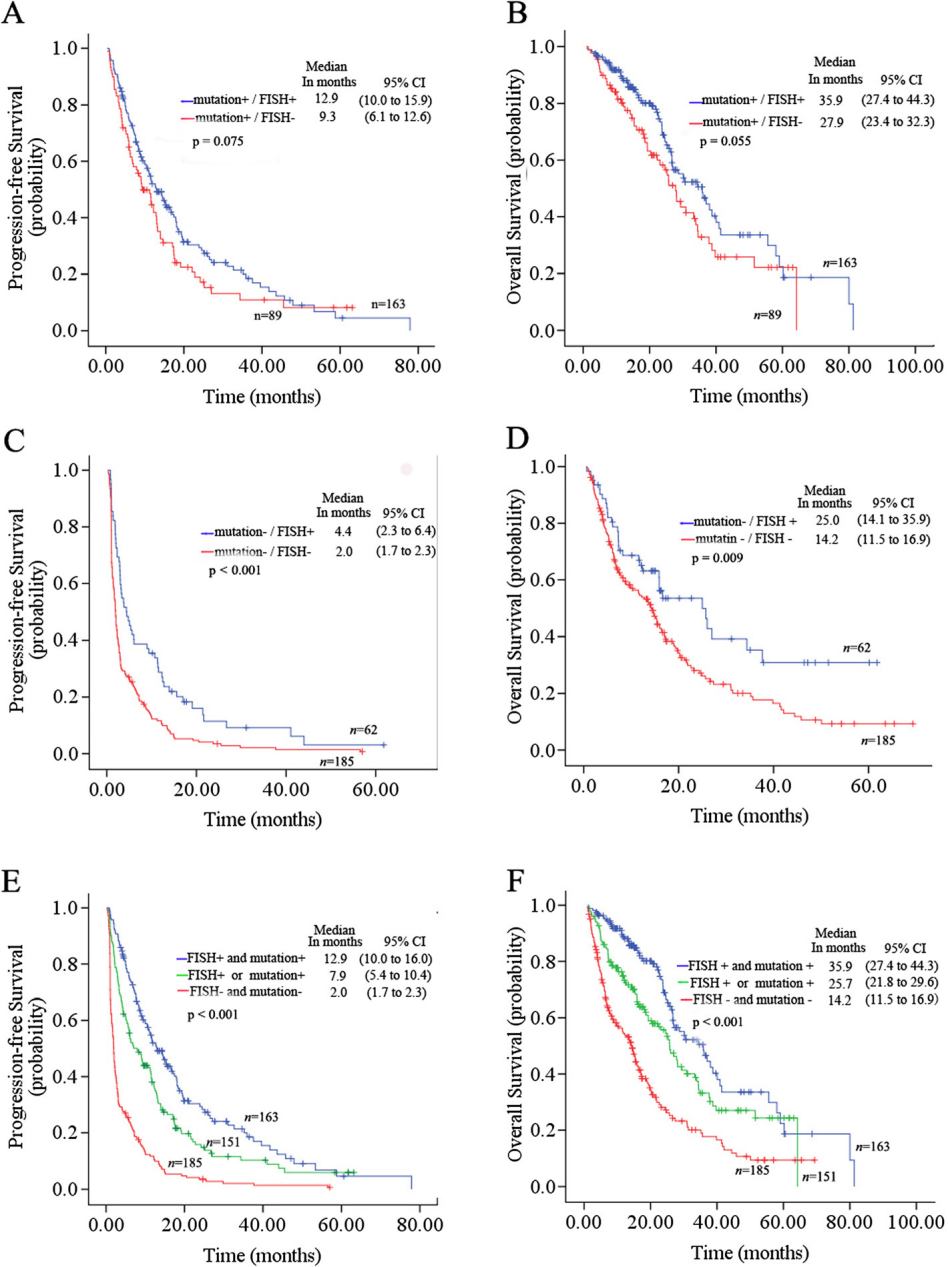
**Kaplan-Meier curves for progression-free survival (PFS) and overall survival (OS) by combined analysis of epidermal growth factor receptor (EGFR) mutation status and EGFR in fluorescent situ hybridization (FISH) status**. (**A**) and (**B**), PFS and OS for FISH status in the subgroup of patients with EGFR mutations; (**C**) and (**D**), PFS and OS for FISH status in the subgroup of patients with wild-type EGFR; (**E**) and (**F**), PFS and OS for double-positive, double-negative, and single-positive EGFR mutations and FISH.

A total of 499 NSCLC cases were available for combined analysis of EGFR gene copy number and EGFR mutations in this study. Among the 252 patients with EGFR mutations, 163 (64.7%) were FISH+; there was no significant association between FISH + and FISH- groups in terms of age, sex, smoking status, and histology (Table 3). There was also no significant improvement in ORR and DCR in mutation+/FISH + patients (p = 0.821 and 0.339, respectively) (Table 1). Moreover, median PFS (12.9 months; 95% CI, 10.0 to 15.9; p = 0.075) and OS (35.9 months; 95% CI, 27.4 to 44.3; p = 0.055) were longer in the mutation+/FISH + group (HR, 0.77; 95% CI, 0.57 to 1.03; p = 0.076 for PFS; HR, 0.70; 95% CI, 0.48 to 1.01; p = 0.057 for OS), but the differences were not significant (Figure 2A, 2B). And improved PFS in patients with mutation+/amplification (HR, 0.72; 95% CI, 0.50 to 1.03; p = 0.073) was not significantly different compared to the patients with mutation+/non-amplification. However, minor superior OS was observed in patients with mutation+/amplification (HR, 0.61; 95% CI, 0.38 to 0.98; p = 0.040).

**Table 3:** Association of EGFR FISH status and patient characteristics in the EGFR mutation stratum

**Characteristics**	**EGFR mutation**			**EGFR wild-type**		
	**FISH + (n = 163)**	**FISH - (n = 89)**	**p**	**FISH+ (n = 62)**	**FISH - (n = 185)**	**p**
Age, years						
Mean (range)	56.7 (29–83)	56.0 (28–80)		57.5 (38–86)	55.9 (23–80)	
Gender			0.822			0.030
Male	83 (50.9)	44 (49.4)		35 (56.5)	132 (71.4)	
Female	80 (49.1)	45 (50.6)		27 (43.5)	53 (28.6)	
Smoking status			0.503			0.743
Non-smoker	120 (73.6)	62 (69.7)		36 (58.1)	103 (55.7)	
Smoker	43 (26.4)	27 (30.3)		26 (41.9)	82 (44.3)	
Histology			0.709			0.635
Adenocarcinoma	144 (88.3)	80 (89.9)		44 (71.0)	137 (74.1)	
Non-adenocarcinoma	19 (11.7)	9 (10.1)		18 (29.0)	48 (25.9)	
Prior platinum			0.426			0.582
Yes	106 (65.0)	57 (64.0)		42 (67.7)	135 (73.0)	
No	53 (32.5)	27 (30.3)		18 (29.0)	42 (22.7)	
Unknown	4 (2.5)	5 (5.6)		2 (3.2)	8 (4.3)	
ECOG PS			0.453			0.454
0, 1	117 (71.8)	62 (69.7)		36 (58.1)	101 (54.6)	
2, 3	35 (21.5)	17 (19.1)		14 (22.6)	56 (30.3)	
Unknown	11 (6.7)	10 (11.2)		12 (19.4)	28 (15.1)	
Disease stage			0.386			0.588
Recurrent	51 (31.3)	35 (39.3)		17 (27.4)	58 (31.4)	
IIIb	27 (16.6)	15 (16.9)		15 (24.2)	34 (18.4)	
IV	85 (52.1)	39 (43.8)		30 (48.4)	93 (50.3)	
EGFR-TKI			0.996			0.845
Gefitinib	119 (73.0)	65 (73.0)		37 (59.7)	113 (61.1)	
Erlotinib	44 (27.0)	24 (27.0)		25 (40.3)	72 (38.9)	
Line of TKI therapy			0.515			0.344
Fist	39 (23.9)	22 (24.7)		11 (17.7)	32 (17.3)	
Second	79 (48.5)	37 (41.6)		32 (51.6)	78 (42.2)	
Higher	45 (27.6)	30 (33.7)		19 (30.6)	75 (40.5)	

Abbreviation: *FISH*, Fluorescent in situ hybridization; *ECOG*, Eastern cooperative oncology group; *PS*, Performance status; *TKI*, Tyrosine kinase inhibitors.

**Figure 3:**
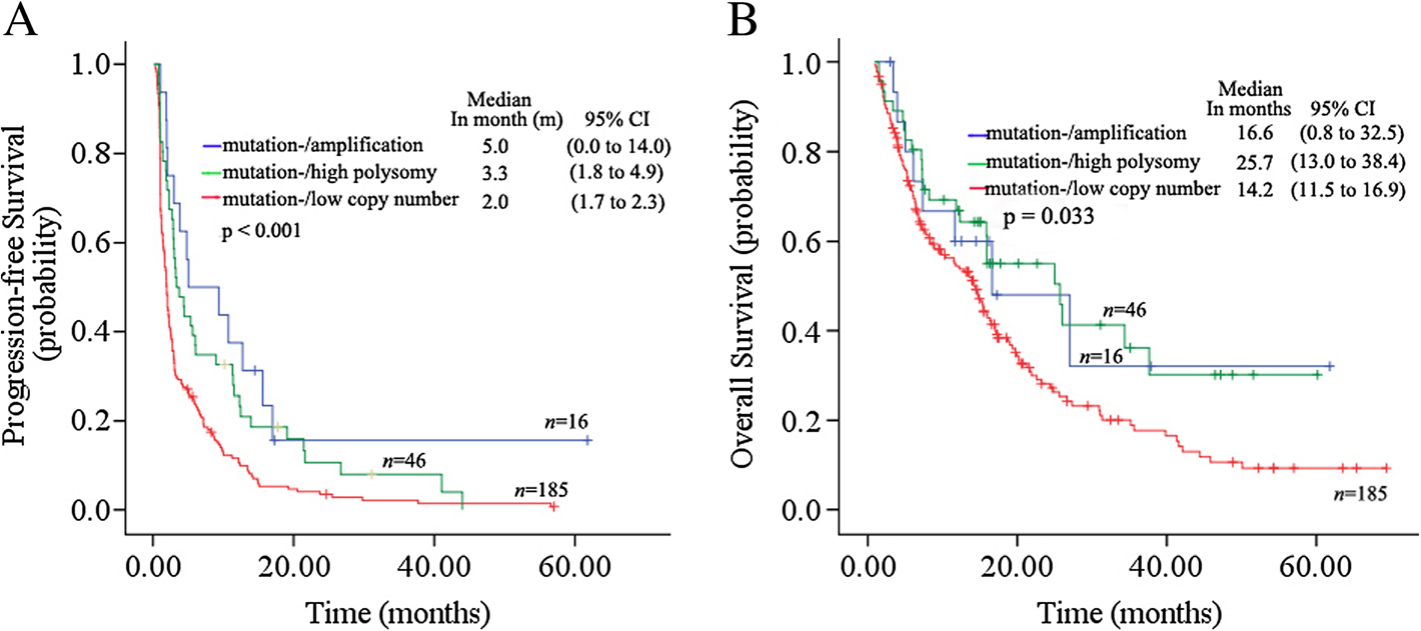
Kaplan-Meier curves for PFS (A) and OS (B) by analysis of epidermal growth factor receptor (EGFR) amplification, high copy number and low copy number in wild-type EGFR.

Among the 247 patients with wild-type EGFR, 62 patients (25.1%) were EGFR-FISH + and were mostly female (p = 0.030); there was no association with age, smoking status, and histology (Table 3). Compared to the mutation-/FISH- subgroup, the mutation-/FISH + patients achieved a significantly higher ORR (17.7% *vs*. 8.6%, p = 0.047), DCR (67.7% *vs*. 35.7%, p < 0.001), and longer PFS (4.4 months *vs*. 2.0 months; HR, 0.56; 95% CI, 0.41 to 0.75; p < 0.001) and OS (25.0 months *vs*. 14.2 months; HR, 0.60; 95% CI, 0.41 to 0.89; p = 0.010) (Table 1, Figure 2C, 2D). Especially, favorable PFS was observed among patients with EGFR amplification compared to low copy number (5.0 months *vs*. 2.2 months; HR, 0.43; 95% CI, 0.24 to 0.76; p = 0.003), but the trend did not affect OS (16.6 months *vs*.15.4 months, HR, 0.65; 95% CI, 0.32 to 1.32; p = 0.228) (Figure 3A, 3B). On the other hand, in two subgroups of EGFR-TKIs, superior PFS (5.6 months *vs*. 2.4 months; HR, 0.59; 95% CI, 0.40 to 0.87; p = 0.007 in gefitinib treatment subgroup, and 3.1 months *vs*. 1.5 months; HR, 0.68; 95% CI, 0.57 to 0.95; p = 0.005 in erlotinib treatment subgroup) could be found in patients with mutation-/FISH + compared to mutation-/FISH-.

Further analysis of the combined markers showed that the 151 patients with either EGFR FISH + status or EGFR mutations (single-positive) had an ORR of 32.5%, a DCR of 79.5%, a median PFS of 7.9 months (95% CI, 5.4 to 10.4), and a median OS of 25.7 months (95% CI, 21.8 to 29.6). Thus, the clinical outcome of patients with EGFR mutation+/FISH + was significantly better than patients with a single-positive mutation and FISH, or those with EGFR mutation-/FISH- (HR for group mutation+/FISH + *vs*. mutation-/FISH-, 0.28; 95% CI, 0.22 to 0.35; p < 0.001; HR for group single-positive *vs*. mutation-/FISH-, 0.41; 95% CI, 0.33 to 0.52; p < 0.001) (Figure 2E, 2F).

## Discussion

To date, there have been controversial reports on the feasibility of using EGFR copy number to predict whether NSCLC patients will benefit from TKI therapy. In a prospective study [14], high EGFR copy number was reported to be significantly associated with a better response, and longer progression-free and overall survival in NSCLC patients treated with gefitinib. Then, the trials (ISEL [19,29] and BR.21 [30]) indicated that EGFR copy number may be a better predictive biomarker for the efficacy of EGFR-TKI than EGFR mutation; but the evidence seemed not much sufficient owing to being placebo-controlled trials. Moreover, EGFR FISH + status failed to predict survival benefits from TKI treatment in the INTEREST [20] and SATURN trials [31]. In the IPASS trial [3], although PFS was significantly longer with gefitinib treatment versus chemotherapy (HR, 0.66; 95% CI, 0.50 to 0.88) in patients with a high EGFR copy number, the authors stated that predictive value was attributed to the high overlap with a coexisting EGFR mutation (77.6% of patients with high EGFR gene copy number harbored a EGFR mutation). A similar conclusion was also presented in the Takano’s report [32], which demonstrated that high EGFR copy numbers are caused by the selective amplification of mutant alleles, according to the discovery of high copy numbers only in patients with a mutant-allele-dominant pattern of mutations. That may contribute to explain why in our study, EGFR FISH + status provided no enhanced ability to predict TKI benefits compared to FISH-, although EGFR FISH + status was an independent predictor of PFS (HR, 0.61; 95% CI, 0.49 to 0.76).

Regardless of EGFR-TKIs used in the first- and second-line settings or maintenance treatment, the presence of mutated-EGFR indicated superior PFS or ORR compared to comparator for therapy of NSCLC in series of trials from the unselected population [20,33] to the superior population [34], and the EGFR mutation-positive population [4,5,7]. Consequently, the overwhelming importance of EGFR mutation status has been established for predicting efficacy and survival benefits from EGFR-TKI.

While attention has been paid to patients with EGFR mutations, closer care should be paid to patients with wild-type EGFR in regard to determining whether or when they should receive EGFR-TKI treatment. The IPASS trial [3] found patients with a high EGFR gene copy numbers had shorter PFS in the absence of an EGFR mutation (HR, 3.85; 95% CI, 2.09 to 7.09). However, a recent study [35] reported that in patients with wild-type EGFR who were diagnosed with squamous cell carcinoma, high EGFR gene copy number correlated well with response to EGFR-TKI therapy (27.3% *vs*. 4.2%, respectively; p = 0.082) and PFS benefit (4.1 months *vs*. 2.1 months, p = 0.201), despite a marginally significant difference due to the sample size. In this study, while comparing the poor outcome of patients who were negative for both of mutations and FISH and had no clinical benefit from EGFR-TKI treatment, we found that patients with FISH+/mutation- obtained favorable DCR and survival benefit. Moreover, the elongated PFS was more marked in the amplification/mutation- subgroup.

We presumed that EGFR FISH status displayed a lower level of predictive sensitivity than mutation. Effect of increased copy number was predominant once EGFR gene was wild-type, no longer playing a full role of prediction. Although evidence of the IPASS [34] and TAILOR [36] trials indicated the superiority of using chemotherapy in a first- and second-line to treat patients who were EGFR mutation-negative, further study is required to verify that EGFR FISH + may favor to choose EGFR-TKIs or chemotherapy treatment in EGFR wild-type population considering improved life-quality and tolerable toxicity.

It is important to note that the variety of subsequent treatments and the difficulty in obtaining detailed information about these subsequent treatments are likely to confound the true outcome of OS. A total of 63 patients (12.5%), among whom 49 had a single-positive mutation and FISH, continued receiving TKIs after diagnosis with advanced disease until the physicians considered tumor progression under control. In this study, we supposed that directed by accumulated clinical experiences, a continued or second application of EGFR-TKIs may potentially contribute to the survival gain in patients harboring EGFR mutations or high copy number.

We are aware that some limitations might be of concern in this study. This is a retrospective study, and alterations in other kinases such as KRAS, BRAF, and cMET that drive tumor growth were not investigated in this study; thus it is possible that the survival and response were affected, at least in part, by those underlying biomarkers. Further prospective, randomized trials are warranted to confirm the efficacy of TKI in NSCLC patients with wild-type EGFR combined with EGFR gene copy number status.

In summary, this study demonstrated that EGFR gene copy number can be further detected in patients with wild-type EGFR, since patients in the FISH + subgroup can derive a greater benefit from EGFR-TKI.

## Abbreviations

NSCLC: Non small cell lung cancer; EGFR: Epidermal growth factor receptor; TKI: Tyrosine kinase inhibitor; FISH: Fluorescence in-situ hybridization; PFS: Progression-free survival; OS: Overall survival; HR: Hazard ratio; ECOG: Eastern cooperative oncology group; PS: Performance status; ORR: Objective response rate; DCR: Disease control rate.

## Competing interests

The authors declare that they have no competing interests.

## Authors’ contributions

SJY conceived and designed the study, finalized the manuscript; WF and FS were responsible for data analysis and interpretation, and drafted the manuscript. SQ carried out the FISH study; ZX, ZX carried out the detection of EGFR mutation analyses; ZWM, ZYB, ZL participated in study design and coordination; XC, LJG, HLX participated in acquiring clinical samples and follow-up clinical information. All authors read and approved the final manuscript.

## Supplementary Material

Additional file 1: Table S1 EGFR Gene Mutation Variables in Luminex Hybridization System. Click here for file

Additional file 2: Table S2 Baseline Characteristics of 502 NSCLC Patients Treated with EGFR-TKIs. Click here for file

Additional file 3: Table S3 Correlation between EGFR FISH Status and Clinicopathological Characteristics of NSCLC Patients. Click here for file
